# Socioeconomic Status and Health: A New Approach to the Measurement of Bivariate Inequality

**DOI:** 10.3390/ijerph14070673

**Published:** 2017-06-23

**Authors:** Guido Erreygers, Roselinde Kessels

**Affiliations:** 1 Department of Economics, University of Antwerp, 2000 Antwerp, Belgium; 2 Centre for Health Policy, University of Melbourne, Melbourne, 3010 Victoria, Australia; 3 Department of Economics, University of Antwerp & Flemish Research Foundation (FWO), 2000 Antwerp, Belgium; roselinde.kessels@uantwerpen.be; 4 Department of Operations Management & Institute for Business and Industrial Statistics, University of Amsterdam, 1018 TV Amsterdam, The Netherlands

**Keywords:** inequality measurement, socioeconomic inequality of health, bivariate inequality, D63, I00

## Abstract

We suggest an alternative way to construct a family of indices of socioeconomic inequality of health. Our indices belong to the broad category of linear indices. In contrast to rank-dependent indices, which are defined in terms of the ranks of the socioeconomic variable and the levels of the health variable, our indices are based on the levels of both the socioeconomic and the health variable. We also indicate how the indices can be modified in order to introduce sensitivity to inequality in the socioeconomic distribution and to inequality in the health distribution. As an empirical illustration, we make a comparative study of the relation between income and well-being in 16 European countries using data from the Survey of Health, Ageing and Retirement in Europe (SHARE) Wave 4.

## 1. Introduction

There is overwhelming empirical evidence that individuals who are relatively well off in economic terms tend to have better health outcomes than those who are less well off. A recent study of the US [1], for instance, revealed that the rich have a much higher life expectancy than the poor. The huge literature on the social gradient in health amply illustrates that inequality in socioeconomic conditions is very often positively and strongly associated to inequality in health achievements (see, e.g., [2,3] and many others).

This paper focuses on the measurement of the extent to which socioeconomic status and health are related to one another. Rank-dependent indicators are by far the most widely used indices to measure socioeconomic inequality of health. The existing rank-dependent indices have been developed to help us find out whether there is pro-rich or pro-poor bias in the health distribution: positive values indicate that people who are relatively well off in socioeconomic terms tend to have better health than those who are less well off, and negative values the opposite. The Concentration Index [4], the extended Concentration Index [5,6], the ‘bounded variable’ indices proposed by Wagstaff [7] and Erreygers [8], and even the Symmetric Index [9] are all indices of this type. In recent years there has been a lively debate on the properties of these indices and on the underlying value judgments (see, e.g., [10,11,12]).

A remarkable feature of rank-dependent indicators is that they measure socioeconomic status by the *ranks* which individuals occupy in the socioeconomic distribution, but health by the *levels* of the health variable under consideration. It could be argued that this asymmetric treatment of socioeconomic status and health reflects a causal relationship between socioeconomic ranks and health levels. The ‘relative position’ hypothesis, however, is only one of several possible explanations of the association between socioeconomic status and health (see, e.g., [13]: Section 5.3). In the absence of any clear evidence on the exact nature of the link between socioeconomic status and health, the choice seems unduly restrictive. There are also no compelling empirical reasons for the asymmetry, because in most cases we do have information on the exact socioeconomic levels which people achieve, e.g., their incomes. Yet rank-dependent indices do not use that information; they exclusively rely on the ranks, not the levels.

Our main aim in this paper is to propose a set of indices based on the levels which individuals attain in the health *and* socioeconomic domains. Indices based on socioeconomic levels capture more information about the socioeconomic distribution than indices based on socioeconomic ranks. We argue that there is a *prima facie* case for the use of information-dense indices, since they give a more complete picture of the socioeconomic distribution, and therefore provide a more accurate measure of socioeconomic inequality of health. A related objective of our paper is to make room for distributional sensitivity. One may have good reasons to be relatively more concerned about those who are not very well off in socioeconomic terms, or who are in particularly bad health. In the literature so far, the focus has been on sensitivity to inequality in the socioeconomic distribution. The extended Concentration Index, for instance, has been constructed by applying suggestions made by Kakwani [14], Donaldson and Weymark [15] and Yitzhaki [16] in the context of income inequality measurement, to rank-dependent indices of socioeconomic inequality. The indices we suggest here can be made sensitive to both the socioeconomic and the health distribution.

Our approach can be summarised as follows. We start from a broad class of indices of bivariate inequality, which encompasses, but is by no means limited to, the set of rank-dependent indices. We specify a limited set of requirements or desired properties for this class of indices, and examine which of these properties our rank-dependent and level-dependent indices possess. We explore how distributional sensitivity can be introduced, and indicate that switching the health and socioeconomic variables opens up a new world of indices. An empirical study using the Survey of Health, Ageing and Retirement in Europe (SHARE) database serves as an illustration.

Since we are dealing with two dimensions, our work is related to the broader literature on multidimensional inequality, such as Tsui [17] and Abul Naga and Geoffard [18]. We concentrate on the measurement of the degree of association between two variables; within this literature, our approach is comparable to that of Zhong [19]. Our set of desired properties depends crucially upon a specific understanding of the notion of correlation. It has been pointed out that the measurement of association relies on implicit value judgements ([20]: 504). In this paper we make our judgements explicit. In contrast to Bommier and Stecklov [20] and Bleichrodt and Van Doorslaer [21] we do not use a social welfare framework. We also do not distinguish fair and unfair inequalities [22].

## 2. Materials and Methods I: Bivariate Linear Indices

### 2.1. Univariate and Bivariate Indices

The indices commonly used in the literature on the measurement of socioeconomic inequality of health can be seen as belonging to a broad class of inequality measures. These indices are closely related to the family of linear measures of inequality defined by Mehran [23]. Mehran focused on the measurement of univariate inequality, say inequality in distribution *x*, where *x* can be income, health, education, or any other variable of interest. Following the representation suggested by Lambert and Lanza [24]: 260–261, we can express the family of univariate linear measures of inequality for discrete distributions as a weighted average of the $x_{i}$ levels:

(1) $$U\left(x\right)=\frac{1}{n}\sum_{i=1}^{n}w_{i}\left(x\right)x_{i}$$

where $w_{i}\left(x\right)$ is the weight of individual *i*, determined in some way by distribution *x*. Typical for rank-dependent measures is that they are characterised by weights which are defined in terms of the ranks of individuals in distribution *x*. There exist, however, many other ways of linking the weights to distribution *x*. Whatever the specific form of the relationship may be, it is customary to assume that the weights always add up to 0:

(2) $$\sum_{i=1}^{n}w_{i}\left(x\right)=0$$

This property ensures that when distribution *x* approaches a perfectly equal distribution, the measured degree of inequality tends to 0. Additional properties, e.g., specifying how the index should react to transfers, can be introduced in order to impose further conditions on the weights.

In this paper we focus on bivariate rather than univariate inequality, and therefore we have to give a slightly different interpretation to the formulas. In the bivariate case, the weights are determined by a different distribution from the one which is weighted. In other terms, the weights are linked to the distribution of a weighting variable, say *z*, which is different from the weighted variable *x*. The Concentration Index, for example, defines the weights in terms of the ranks of individuals in the socioeconomic distribution, and applies these weights to the distribution of health. In formal terms, the bivariate linear index where *z* serves as the weighting variable and *x* as the weighted variable can be defined as:

(3) $$B\left(z,x\right)=\frac{1}{n}\sum_{i=1}^{n}w_{i}\left(z\right)x_{i}$$

Expressions (1) and (3) concern the so-called absolute versions of the univariate and bivariate indices. We refer to Kolm [25,26] for a detailed discussion of absolute and relative inequality. The relative versions are obtained by dividing the indices by the mean of the weighted variable, $\mu_{x}$. The relative version of the univariate index is therefore $U^{*}\left(x\right)=\frac{1}{\mu_{x}}U\left(x\right)$, and that of the bivariate index $B^{*}\left(z,x\right)=\frac{1}{\mu_{x}}B\left(z,x\right)$. Other versions of the indices have been proposed in the literature, e.g., in order to deal with bounded variables [7,8]. Erreygers and Van Ourti [27] have argued that the choice for a specific version of a bivariate index should be made in accordance with the type of weighted variable under consideration. We henceforth assume that both the weighted and the weighting variables are nonnegative ratio-scale variables.

We will now explore what kind of conditions can reasonably be imposed upon the weights $w_{i}\left(z\right)$.

### 2.2. Properties of Bivariate Linear Indices

Bivariate indices of inequality serve another purpose than univariate indices of inequality. While univariate indices measure the dispersion or spread of a distribution, bivariate indices are intended as measures of the degree of association or correlation between two distributions. In statistical terms, the difference may be thought of as corresponding to that between the variance (of one variable) and the covariance (between two variables). When examining whether a particular bivariate linear index is acceptable or not, we therefore have to use a checklist of desirable properties which relate to the correlation between the variables under consideration.

We adopt a pedestrian approach in order to avoid drowning the reader in a sea of formulas. We begin by enouncing a number of properties for which we think a reasonable case can be made. When compiling our list of properties we have taken inspiration from axiomatic approaches to multidimensional inequality [17,18]. However, since we concentrate on the association between two variables and limit ourselves to the class of bivariate linear indices, our focus is more narrow. The aim is to obtain meaningful restrictions on the weights $w_{i}\left(z\right)$. After enouncing the properties, we therefore explore which conditions these properties imply for the weights.

#### 2.2.1. Five Properties

The properties we would like our indices to have relate to the following aspects: the information taken into consideration to determine the weights; the measurement unit of the variable used to determine the weights; the evaluation of cases of clearly uncorrelated variables; the direction of change in reaction to increases in correlation; and the nature of this change.

**Anonimity**: *Individuals who are identical with respect to the weighting variable, have identical weights.*

The motivation is that the weights should be determined by nothing else but the weighting variable. For instance, if income is used as the weighting variable, other dimensions such as age and sex are irrelevant for the determination of the weights.

**Unit consistency**: *The weights are independent of the specific unit in which the weighting variable is measured.*

Unit consistency is a common requirement when it comes to measuring inequality (see, e.g., [28]). For instance, if incomes are used to determine the weights, we want our weights to be the same whether we are using $ or € to measure incomes.

**Neutrality**: *If either the weighting or the weighted variable is perfectly equally distributed, the measured degree of bivariate inequality is zero.*

The main function of this property is to allow us to define the borderline between cases of positive correlation, indicated by positive values of the index, and cases of negative correlation, indicated by negative values. With regard to socioeconomic inequality of health, for instance, a positive correlation means that there is a pro-rich bias in the distribution of health, and a negative correlation that there is a pro-poor bias. Ideally, the value of the index should be zero in all cases where the variables are uncorrelated, not just in the two cases mentioned above. To check that, we need a precise definition of what we mean by two variables being uncorrelated. We do not provide such a definition here, and limit ourselves to the consideration of the effects of the two special cases.

**Coherence**: *Any change which definitely increases the positive (c.q. negative) correlation between the weighting and the weighted variable, leads to a positive (c.q. negative) change of the measured degree of bivariate inequality.*

The idea is to ensure that changes which undoubtedly increase the positive (c.q. negative) correlation between two variables should be reflected coherently by the index. Below we will make use of position switches between individuals to characterise changes which increase the positive (c.q. negative) correlation between the two variables. A more elaborate discussion of bivariate correlation can be found in [29].

**Continuity**: *If the distribution of either the weighting or the weighted variable is changed in a given direction, the measured degree of bivariate inequality changes continuously.*

This property expresses a preference for indices that change smoothly rather than spasmodically.

#### 2.2.2. Anonimity, Unit Consistency and Neutrality

The conditions imposed by the Anonimity property are immediately clear. When two individuals *i* and *j* are identical with respect to the weighting variable, then their weights must be the same:

(4) $$z_{i}=z_{j}⟹w_{i}\left(z\right)=w_{j}\left(z\right)$$

What is required for Unit consistency depends upon the nature of the weighting variable. When *z* is a ratio-scale variable, as we assume in this paper, the weights should be independent of the scale, i.e.,:

(5) $$\forall \lambda >0:w_{i}\left(z\right)=w_{i}\left(\lambda z\right)\;\;\;\;\;i=1,2,\ldots ,n$$

The implications of the Neutrality property can be derived from two simple tests. First we assume that the weighted variable is a constant no matter what the level of the weighting variable is, and second that the weighting variable is a constant no matter what the level of the weighted variable is. In both cases we have to find out under which conditions the value of the index is zero. Let $\bar{x}$ and $\bar{z}$ be perfectly equal distributions, i.e., characterised by respectively $x_{1}=x_{2}=\ldots =x_{n}=\mu_{x}$ and $z_{1}=z_{2}=\ldots =z_{n}=\mu_{z}$. An index passes the first test if $B(z,\bar{x})=0$, whatever *z* may be. Since $B\left(z,\bar{x}\right)=\frac{1}{n}\mu_{x}\sum_{i=1}^{n}w_{i}\left(z\right)$, the first test leads to the condition that the sum of the weights must be zero:

(6) $$\forall z:\sum_{i=1}^{n}w_{i}\left(z\right)=0$$

In order to pass the second test, the index must be such that $B(\bar{z},x)=0$, whatever *x* may be. This leads to the condition:

(7) $$\forall \bar{z}:\sum_{i=1}^{n}w_{i}\left(\bar{z}\right)=0$$

Clearly, condition (7) is implied by condition (6).

Observe that if the Anonimity property holds, a perfectly equal distribution $\bar{z}$ implies equal weights for all individuals. This means that Anonimity and Neutrality together imply that condition (7) can be sharpened to the condition that in case of equality all the weights must equal 0:

(8) $$\forall \bar{z},\forall i=1,2,\ldots ,n:w_{i}\left(\bar{z}\right)=0$$

#### 2.2.3. Coherence

It is a bit more complicated to derive the conditions implied by the Coherence property. We have to begin by specifying what we mean by changes which definitely increase the positive (c.q. negative) correlation between variables. First we look at changes in the *x* space. Let us choose an arbitrary individual *i*, with values $\left(z_{i},x_{i}\right)$, as our reference person. We then select another person *j*, with values $\left(z_{j},x_{j}\right)$, such that $x_{i}\neq x_{j}$ and $z_{i}\neq z_{j}$. The idea is to switch the positions of persons *i* and *j* in the *x* space, i.e., to suppose that person *i* obtains level $x_{j}$ rather than $x_{i}$, and person *j* level $x_{i}$ rather than $x_{j}$. This is equivalent to a ‘transfer’ $\delta_{x}=x_{j}-x_{i}$ in the *x* space between persons *j* and *i*. Two cases must be distinguished, $z_{i}<z_{j}$ and $z_{i}>z_{j}$. In the first case, if initially we have $x_{i}<x_{j}$, the switch can be seen as increasing the negative correlation between *x* and *z*, because after the transfer we have $x_{i}+\delta_{x}>x_{j}-\delta_{x}$, which means that the relative positions of *i* and *j* in the *x* space are now different from what they are in the *z* space. This is what we call a Negative-Correlation-Enhancing (NCE) transfer in the *x* space. If, however, we have $x_{i}>x_{j}$, the switch can be seen as increasing the positive correlation between *x* and *z*, because after the transfer we have $x_{i}+\delta_{x}<x_{j}-\delta_{x}$. This is a Positive-Correlation-Enhancing (PCE) transfer in the *x* space. In the second case, exactly the opposite holds: if initially we have $x_{i}<x_{j}$, the switch leads to a PCE transfer in the *x* space, and if we had $x_{i}>x_{j}$, to a NCE transfer.

We can just as well look at changes in the *z* space. Let us consider a switch between persons *i* and *j* corresponding to a transfer $\delta_{z}=z_{j}-z_{i}$ in the *z* space between persons *j* and *i*. Following the same reasoning as before, we say that if we have $x_{i}<x_{j}$ and $z_{i}<z_{j}$ or $x_{i}>x_{j}$ and $z_{i}>z_{j}$, the switch constitutes a NCE transfer in the *z* space, and if we have $x_{i}>x_{j}$ and $z_{i}<z_{j}$ or $x_{i}<x_{j}$ and $z_{i}>z_{j}$, a PCE transfer in the *z* space.

We are now in a position to assess the implications of the Coherence property. Let us begin by position switches in the *x* space. The transfer $\delta_{x}$ affects individuals *i* and *j* only. The change in the value of the index is therefore equal to $\delta_{x}\left[w_{j}\left(z\right)-w_{i}\left(z\right)\right]$. Obviously, the value of the index increases if and only if $\delta_{x}$ and $w_{j}\left(z\right)-w_{i}\left(z\right)$ have the same sign, and it decreases if and only if $\delta_{x}$ and $w_{j}\left(z\right)-w_{i}\left(z\right)$ have opposite signs. Hence, when it comes to changes in the *x* space, a PCE transfer increases the value of the index and a NCE transfer decreases it if and only if the weights are increasing in the weighting variable:

(9) $$z_{i}<z_{j}⟹w_{i}\left(z\right)<w_{j}\left(z\right)$$

Next, we look at position switches in the *z* space. First we have to check whether there is any change in the weights, and second we have to see how they change. If there is no change in the weights, the value of the index remains the same. In that case, the index does not have the Coherence property. If the weights do change, three cases must be distinguished. To begin with, suppose the transfer $\delta_{z}$ changes the weight of one individual only, say that of individual *k* (which may be *i*, *j* or any other). Let us denote the difference in the weight of *k* by $\Delta w_{k}$. The change in the value of the index is then equal to $x_{k}\Delta w_{k}$. In the case of a PCE transfer we must have $x_{k}\Delta w_{k}>0$, and in that of a NCE transfer $x_{k}\Delta w_{k}<0$. Since we cannot both have $\Delta w_{k}>0$ and $\Delta w_{k}<0$, the Coherence property cannot hold. Next, let us assume that the transfer affects the weights of just two individuals, *k* and *l* (which may be *i*, *j* or any other). The differences in the weights are now $\Delta w_{k}$ and $\Delta w_{l}$, and the change in the value of the index is equal to $x_{k}\Delta w_{k}+x_{l}\Delta w_{l}$. In the case of a PCE transfer we must have $x_{k}\Delta w_{k}+x_{l}\Delta w_{l}>0$, and in that of a NCE transfer $x_{k}\Delta w_{k}+x_{l}\Delta w_{l}<0$, which is possible only if one of the differences is positive and the other negative. Let us assume that $\Delta w_{l}>0$. Then the index reacts coherently to a PCE transfer if and only if $-\Delta w_{k}/\Delta w_{l}<x_{l}/x_{k}$ and coherently to a NCE transfer if and only if $-\Delta w_{k}/\Delta w_{l}>x_{l}/x_{k}$. That leaves just one possibility: $\left\{k,l\right\}=\left\{i,j\right\}$ and $-\Delta w_{i}=\Delta w_{j}$. Finally, if three or more weights are affected, it is impossible to impose conditions on the weights which ensure that the index reacts positively to a PCE transfer and negatively to a NCE transfer. Hence, we arrive at the conclusion that as far as position switches in the *z* space are concerned, three conditions must hold: (i) a switch must always affect some weights; (ii) a switch must affect only the weights of the persons involved in the switch; and (iii) the changes in the weights must be of equal magnitude but of opposite sign.

This has strong implications for the weights. In fact, it follows that the weight $w_{i}\left(z\right)$ of any individual *i* is a function only of this individual’s $z_{i}$ level and possibly of the average $\mu_{z}$, i.e., $w_{i}\left(z\right)=w_{i}\left(\mu_{z},z_{i}\right)$. Moreover, for a transfer $\delta_{z}=z_{j}-z_{i}$ between *j* and *i* to always lead to $-\Delta w_{i}=\Delta w_{j}$ whatever the size of $\delta_{z}$ and whoever *j* might be, the weight of any individual *i* must be a linear function of $z_{i}$:

(10) $$\forall i=1,2,\ldots ,n:w_{i}\left(\mu_{z},z_{i}\right)=\alpha_{i}\left(\mu_{z}\right)+\beta \left(\mu_{z}\right)z_{i}$$

with $\beta (\mu_{z})>0$. It is easy to check that in this case we have: $-\Delta w_{i}=\beta \left(\mu_{z}\right)\delta_{z}=\Delta w_{j}$.

We can go a little bit further by assuming that Anonimity and Neutrality also hold. Anonimity implies that $\alpha_{i}\left(\mu_{z}\right)=\alpha_{j}\left(\mu_{z}\right)$ for any *i* and *j*, otherwise (4) would be violated. Hence, we have:

(11) $$\forall i=1,2,\ldots ,n:w_{i}\left(\mu_{z},z_{i}\right)=\alpha \left(\mu_{z}\right)+\beta \left(\mu_{z}\right)z_{i}$$

which means in fact that all $w_{i}\left(\mu_{z},z_{i}\right)$ functions are identical, and we can write $w_{i}\left(z\right)=w\left(\mu_{z},z_{i}\right)$. Neutrality implies that the sum of all the weights must be zero. Combining (6) and (11), we find that $\alpha \left(\mu_{z}\right)=-\beta \left(\mu_{z}\right)\mu_{z}$, and therefore that:

(12) $$\forall i=1,2,\ldots ,n:w\left(\mu_{z},z_{i}\right)=\beta \left(\mu_{z}\right)\left[z_{i}-\mu_{z}\right]$$

In other words, the weights $w_{i}\left(z\right)$ must be proportional to the deviations of the $z_{i}$ levels from the average $\mu_{z}$.

#### 2.2.4. Continuity

We now turn to the Continuity property. An index should react smoothly to given changes in both the *x* space and the *z* space. We begin by looking at changes in the *x* space. Let *x* and $x^{\prime }$ be two different distributions, and consider the linear combination $x\left(\phi \right)=\phi x+\left(1-\phi \right)x^{\prime }$, with 0≤ϕ≤1. Since $x\left(\phi \right)=x+\left(1-\phi \right)\left(x^{\prime }-x\right)$, it follows that x(ϕ) is obtained by changing *x* in the direction $\left(x^{\prime }-x\right)$. We have to see how B(z,x(ϕ)) changes when ϕ goes from 1 to 0. Since $B\left(z,x\left(\phi \right)\right)=\phi B\left(z,x\right)+\left(1-\phi \right)B\left(z,x^{\prime }\right)$, it is clear that the value of the index changes continuously and tends to $B(z,x^{\prime })$ when ϕ goes from 1 to 0. Hence, the index always reacts continuously to changes in the *x* space. Next, we look at changes in the *z* space. Let *z* and $z^{\prime }$ be two different distributions, and consider the linear combination $z\left(\phi \right)=\phi z+\left(1-\phi \right)z^{\prime }$. Since $B\left(z\left(\phi \right),x\right)=\frac{1}{n}\sum_{i=1}^{n}w_{i}\left(z\left(\phi \right)\right)x_{i}$, it follows that the weights $w_{i}\left(z\left(\phi \right)\right)$ must change continuously and tend to $w(z^{\prime })$ when ϕ goes from 1 to 0. For instance, if we had $w_{i}\left(z\left(\phi \right)\right)=\phi w_{i}\left(z\right)+\left(1-\phi \right)w_{i}\left(z^{\prime }\right)$, then we would have $B\left(z\left(\phi \right),x\right)=\phi B\left(z,x\right)+\left(1-\phi \right)B\left(z^{\prime },x\right)$ and the condition would be satisfied. However, there are many other possible ways in which the weights can change.

## 3. Materials and Methods II: Indices of Socioeconomic Inequality of Health

### 3.1. Socioeconomic Status and Health

From now on we focus on the measurement of socioeconomic inequality of health. Suppose we have information on the socioeconomic achievement ($y_{i}$) and the health attainment ($h_{i}$) of each individual *i* in a population consisting of *n* individuals (i=1,2,...,n). To simplify things, we assume that both the socioeconomic variable *y* and the health variable *h* are ratio-scale variables with well-defined lower bounds greater than or equal to 0. Moreover, each of these variables has either no upper bound or a well-defined upper bound. The socioeconomic and health variables are not necessarily of the same nature. Typically, the socioeconomic variable is an unbounded variable, e.g., income or consumption; this is also the assumption adopted in our paper. The health variable, by contrast, is often a bounded variable, e.g., a measur;een 0% and 100%. The means of the variables are $\mu_{y}=\frac{1}{n}\sum_{i=1}^{n}y_{i}$ and $\mu_{h}=\frac{1}{n}\sum_{i=1}^{n}h_{i}$, respectively.

Given the levels of the socioeconomic and health variables, we can determine the ranks of all individuals in the socioeconomic and health distributions. We denote the rank of individual *i* in the socioeconomic distribution as $r_{i}$ and in the health distribution as $q_{i}$. If there are no ties in a distribution, the rank of the person with the lowest level is equal to 1, the rank of the person with the second lowest level equal to 2, etc., and the rank of the person with the highest level equal to *n*. If a group of k+1 individuals are tied on position *g*, the rank of each of these individuals is equal to g+(k/2). For simplicity, however, we assume there are no ties in the distributions. This assumption is made in order to simplify the expressions of the rank-dependent indices; it is by no means necessary.

### 3.2. Rank-Dependent Indices

As already mentioned, rank-dependent indices are special cases of linear indices. Given a weighting variable such as income or socioeconomic status, we use the rank of person *i* in the distribution of the weighting variable to determine the weight of person *i*. The level of person *i* in this distribution serves only to derive the rank of that person; it does not enter directly into the calculation of the weight. The standard Concentration Index, for instance, is characterised by a weighting function which is linear in the ranks $r_{i}$ determined by the socioeconomic distribution *y*. The weighting function may therefore be called the ‘socioeconomic rank’ function and the associated index the SR index:

(13) $$w_{i}\left(y\right)=w_{i}^{SR}=\frac{2r_{i}-n-1}{n}$$

The weights steadily increase as $r_{i}$ goes from 1 to *n*. If we define the group of the ‘poor’ as those who have negative weights and the group of the ‘rich’ as those who have positive weights, the weighting function (13) puts the boundary between the two groups exactly in the middle of the population. Those with ranks smaller than or equal to n/2 (if *n* is even) or smaller than or equal to (n−1)/2 (if *n* is odd) have negative weights, and those with ranks larger than or equal to n/2+1 (if *n* is even) or larger than or equal to (n+1)/2 (if *n* is odd) have positive weights.

### 3.3. Assessing Rank-Dependent Indices

Rank-dependent indices focus on the ranks of individuals in the distribution of the weighting variable, and hence use only part of the available information on this variable. Strictly speaking, rank-dependent indices capture the correlation between the *ranks* of the weighting variable and the *levels* of the weighted variable rather than the correlation between the *levels* of the weighting variable and the *levels* of the weighted variable. In order to see whether this constitutes a drawback, we have to check whether rank-dependent indices possess the properties introduced in the previous section. We now show the following result.

**Theorem** **1.** *The rank-dependent index SR has the properties of Anonimity, Unit consistency and Neutrality, but not those of Coherence and Continuity.*

**Proof.**  (i) Anonimity. Individuals who attain the same level in the socioeconomic domain have the same socioeconomic rank and therefore the same weight: $y_{i}=y_{j}⟹r_{i}=r_{j}⟹w_{i}^{SR}=w_{j}^{SR}$; (ii) Unit consistency. Multiplying all socioeconomic levels by a factor λ>0 leaves all ranks unchanged, which means that all the weights $w_{i}^{SR}$ remain the same; (iii) Neutrality. We always have $\sum_{i=1}^{n}w_{i}^{SR}=0$; moreover, in the case of perfect equality in the socioeconomic domain, we have $r_{1}=r_{2}=\ldots =r_{n}=\frac{n+1}{2}$ and therefore $w_{1}^{SR}=w_{2}^{SR}=\ldots =w_{n}^{SR}=0$; (iv) Coherence. Since we have $y_{i}<y_{j}⟹r_{i}<r_{j}⟹w_{i}^{SR}<w_{j}^{SR}$, the weights are increasing in the weighting variable, and therefore rank-dependent indices react coherently to transfers in the *h* space. However, the same does not hold with regard to transfers in the *y* space. In fact, the weights are functions of the ranks rather than the levels of the weighting variable. That means that condition (12) is not satisfied, unless the ranks are proportional to the levels, which happens only by fluke. A given transfer in the *y* space can therefore have a large or a small effect, depending on the density of the distribution in the region where the transfer takes place; (v) Continuity. If the socioeconomic distribution *y* is changed gradually to the distribution $y^{\prime }$ following the trajectory $\phi y+\left(1-\phi \right)y^{\prime }$, the ranks of individuals do not change smoothly, but by means of (small) bumps. For a change in ranks to occur, previously different individuals ($y_{i}\neq y_{j}$) must become tied ($\phi y_{i}+\left(1-\phi \right)y_{i}^{\prime }=\phi y_{j}+\left(1-\phi \right)y_{j}^{\prime }$). As long as the ranks remain the same, the value of the index does not change. Hence, SR does not change smoothly when the distribution of the weighting variable is changed in a given direction. ☐

The explanation of this result lies in the fact that the ranks of individuals say very little about the levels which these individuals attain, and that the difference between the ranks of two individuals does not tell us very much about about the difference in the levels which these individuals attain. The levels and their differences may be very large, very small, or something in between. The challenge, therefore, is to find a way to construct an indicator which does take the levels of the weighting variable into account in the determination of the weights. Put differently, we want to explore whether and how an indicator can be based on level-dependent rather than rank-dependent weights.

### 3.4. Indices Based on Socioeconomic Levels

We now introduce an alternative way to define the individual weights. Instead of letting the weights depend exclusively on the ranks of individuals, we define them in terms of the levels of the socioeconomic variable. In other words, we work with a weighting function, the arguments of which are levels, not ranks. What we are looking for is a set of individual weights which satisfy the conditions implied by the five properties mentioned earlier.

Looking at (12), it is obvious that the weights $w_{i}$ must be proportional to the deviations of the socioeconomic levels $y_{i}$ from the mean socioeconomic level $\mu_{y}$, i.e., proportional to $d_{i}=y_{i}-\mu_{y}$. What remains to be decided is the shape of the proportionality function $\beta (\mu_{y})$. The property of Unit consistency requires that this function is homogeneous of degree −1 in $\mu_{y}$. In fact, if all $y_{i}$ are multiplied by the factor λ>0, all deviations are also multiplied by the factor λ. That implies that the weights remain unchanged only if $\beta (\mu_{y})$ changes by the factor $\lambda^{-1}$. The simplest function of this type is:

(14) $$\beta \left(\mu_{y}\right)=\frac{1}{\mu_{y}}$$

This functional form leads to the following ‘socioeconomic level’ weighting scheme:

(15) $$w_{i}^{SL}=\frac{y_{i}-\mu_{y}}{\mu_{y}}$$

Observe that it is possible to rewrite (15) as the product of the two terms: the ratio of the deviation $d_{i}$ to the absolute mean deviation $\mu_{\left|d\right|}=\frac{1}{n}\sum_{i=1}^{n}\left|d_{i}\right|$, and the ratio of the absolute mean deviation $\mu_{\left|d\right|}$ to the mean $\mu_{y}$. This decomposes the weight into two components: one part which measures the relative position of individual *i* by the normalised deviation ($d_{i}/\mu_{\left|d\right|}$) of this person, and another which represents the degree of inequality in distribution *y* as measured by the relative mean deviation $\mu_{\left|d\right|}/\mu_{y}$. An alternative expression for the weighting function of the socioeconomic level-dependent index SL is therefore:

(16) $$w_{i}^{SL}=\frac{d_{i}}{\mu_{\left|d\right|}}.\frac{\mu_{\left|d\right|}}{\mu_{y}}=\frac{y_{i}-\mu_{y}}{\frac{1}{n}\sum_{j=1}^{n}\left|y_{j}-\mu_{y}\right|}.\frac{\frac{1}{n}\sum_{j=1}^{n}\left|y_{j}-\mu_{y}\right|}{\mu_{y}}$$

Note that the negative weights sum to $-\left(n/2\right)\left(\mu_{\left|d\right|}/\mu_{y}\right)$, and the positive weights to $\left(n/2\right)\left(\mu_{\left|d\right|}/\mu_{y}\right)$.

### 3.5. Assessing Level-Dependent Indices

Do level-dependent indices perform better than rank-dependent ones? We have the following result.

**Theorem** **2.** *The level-dependent index SL has the properties of Anonimity, Unit consistency, Neutrality, Coherence and Continuity.*

**Proof.**  (i) Anonimity. It is easy to check that we have $y_{i}=y_{j}⟹w_{i}^{SL}=w_{j}^{SL}$; (ii) Unit consistency. Multiplying all socioeconomic levels by a factor λ>0 has no effect on the weights $w_{i}^{SL}$; (iii) Neutrality. We always have $\sum_{i=1}^{n}w_{i}^{SL}=0$, and if $y_{1}=y_{2}=\ldots =y_{n}$, then we have $w_{1}^{SL}=w_{2}^{SL}=\ldots =w_{n}^{SL}=0$; (iv) Coherence. This is implied by the fact that the weighting function (15) is consistent with (12); (v) Continuity. If we go from *y* to $y^{\prime }$ following the trajectory $y\left(\phi \right)=\phi y+\left(1-\phi \right)y^{\prime }$, the weights change according to the formula:

$$w_{i}^{SL}\left(y\left(\phi \right)\right)=\frac{\phi \mu_{y}}{\phi \mu_{y}+\left(1-\phi \right)\mu_{y^{\prime }}}w_{i}^{SL}\left(y\right)+\frac{\left(1-\phi \right)\mu_{y^{\prime }}}{\phi \mu_{y}+\left(1-\phi \right)\mu_{y^{\prime }}}w_{i}^{SL}\left(y^{\prime }\right)$$

which means that the value of the level-dependent index changes smoothly. Note that in the special case where *y* and $y^{\prime }$ have the same mean, we obtain: $w_{i}^{SL}\left(y\left(\phi \right)\right)=\phi w_{i}^{SL}\left(y\right)+\left(1-\phi \right)w_{i}^{SL}\left(y^{\prime }\right)$, which implies that the index changes in a simple linear way. ☐

If we believe it is important that indices of socioeconomic inequality of health possess the five properties of Anonimity, Unit consistency, Neutrality, Coherence and Continuity, then it is clear level-dependent indices must be preferred over rank-dependent ones.

### 3.6. Extended Indices

Before we turn our attention to an empirical application, we explore two possible adaptations of the rank- and level-dependent indices introduced above. The first concerns modifications aimed at making indices more sensitive to what happens at the bottom of the distribution. The second consists of inverting the health and socioeconomic variables.

#### 3.6.1. Distributional Sensitivity

Both in the literature on univariate inequality and in that on bivariate inequality, indices have been developed which react differently to changes occurring at different locations in the distribution of the weighting variable. When measuring income inequality, for instance, one often uses the extended Gini coefficient [14,15,16], the Atkinson index [30] or the generalised entropy index [31], all of which include a parameter allowing the expression of varying degrees of inequality aversion. In the context of socioeconomic inequality of health, we have the similar concept of the extended Concentration Index [5,6].

The idea behind distributional sensitivity is that a given change in the distribution of the weighted variable (e.g., a ‘transfer’ of health among two individuals) should have a different effect on the value of the index depending on where the individuals concerned by the change are located in the distribution of the weighting variable (e.g., a transfer among the poor should have a greater effect than one among the rich). In line with what is customary in the literature on economic inequality (see [26] and more recently [32]), our focus here is on giving more weight to changes occurring at the lower end of the distribution. Using the notion of position switches, we now introduce the following property.

**Sensitivity**: *A given position switch in the space of the weighted variable has a larger absolute impact when it occurs at the lower end of the distribution of the weighting variable than when it occurs at the higher end.*

Let us look at position switches of equal magnitude in the *x* space between different pairs of people who are all at equal distance in the distribution of the weighting variable *z*. Given that a transfer $\delta_{x}=x_{j}-x_{i}$ between persons *j* and *i* changes the value of the index by $\delta_{x}\left[w_{j}\left(z\right)-w_{i}\left(z\right)\right]$, the magnitude of the effect is proportional to the difference between the weights assigned to *i* and *j*. The effect of the transfer $\delta_{x}$ is sensitive to the distribution of the weighting variable if and only if this difference depends on the location of *i* and *j* in distribution *z*. More specifically, the absolute difference between the weights of equidistant individuals must become smaller and smaller as one moves from the low end to the high end of distribution *z*. In formal terms, this means the property of Sensitivity holds if and only if:

(17) $$\left\{z_{j}-z_{i}=z_{l}-z_{k}>0,z_{i}<z_{k}\right\}⟹\left|w_{j}\left(z\right)-w_{i}\left(z\right)\right|>\left|w_{l}\left(z\right)-w_{k}\left(z\right)\right|$$

Before we see how both rank-dependent and level-dependent indices can be made distributionally sensitive, it seems useful to examine whether the property of Sensitivity can be reconciled with the other properties we have introduced. Unfortunately, this is not the case.

**Theorem** **3.** *Bivariate linear indices which have the properties of Anonimity and Neutrality, cannot simultaneously have the properties of Coherence and of Sensitivity.*

**Proof.**  This follows immediately from a comparison of conditions (12) and (17). For any $z_{j}-z_{i}=z_{l}-z_{k}$, condition (12) implies $w_{j}\left(z\right)-w_{i}\left(z\right)=w_{l}\left(z\right)-w_{k}\left(z\right)$, and hence condition (17) cannot be satisfied. ☐

While Coherence imposes a linear weighting scheme, Sensitivity imposes a nonlinear one. The implication is that we have to choose between Coherence and Sensitivity: we simply cannot have both.

To end our discussion of distributional sensitivity, we would like to point out that the concept need not be limited to the lower end of the distribution. Erreygers, Clarke and Van Ourti [9] have made a case for sensitivity to changes occurring at both ends of the distribution. However, what they have called the symmetry property can be defined only if the weighting variable follows a uniform distribution. Since this is in general not the case, we limit ourselves to the case of lower-end distributional sensitivity.

#### 3.6.2. Extended Rank-Dependent Indices

Distributional sensitivity has been introduced for rank-dependent indices by transforming the weighting function (13), which is linear in the ranks, into a nonlinear one. The weighting function of the extended Concentration Index has been developed to give relatively more weight to individuals with lower ranks. Slightly generalizing the approach of Erreygers, Clarke and Van Ourti [9], the extended version of the SR weighting function can be expressed as follows:

(18) $$w_{i}^{SR}\left(\nu \right)=\left\{\begin{array}{c} \frac{1+n\left[{\left(\frac{n-r_{i}}{n}\right)}^{\nu }-{\left(\frac{n-r_{i}+1}{n}\right)}^{\nu }\right]}{\nu -1},\;\;\;\;\;\nu >0,\nu \neq 1\\ \left(n-r_{i}\right)\ln\left(\frac{n-r_{i}}{n}\right)-\left(n-r_{i}+1\right)\ln\left(\frac{n-r_{i}+1}{n}\right),\;\;\;\;\;\nu =1 \end{array}\right.$$

where ν is a distributional sensitivity parameter. The extended SR index is denoted by SR(ν). The linear weighting function (13) corresponds to the value ν=2. The more the value of ν exceeds this threshold, the more sensitive the index is to changes in the lower end of the rank distribution. The sum of the negative weights is equal to $-n/(\nu^{\nu /(\nu -1)})$, and that of the positive weights $n/(\nu^{\nu /(\nu -1)})$. For ν>1, the lowest negative weight is (approximately) −1, and the highest positive weight (approximately) 1/(ν−1).

#### 3.6.3. Extended Level-Dependent Indices

For level-dependent indices, distributional sensitivity requires an adaptation of the linear weighting scheme (15). We will do so by changing one of the components of the equivalent expression (16). One way of making the weights more sensitive to the bottom of the socioeconomic distribution consists of first applying a transformation to the socioeconomic levels. A convenient parametric form is of the isoelastic type:

(19) $$y_{i}\left(\alpha \right)=\left\{\begin{array}{cc} \frac{y_{i}^{1-\alpha }-\alpha }{1-\alpha } & \left(\alpha \neq 1\right)\\ 1+\ln(y_{i}) & \left(\alpha =1\right) \end{array}\right.$$

In order to make sure that $y_{i}\left(\alpha \right)$ exists for α≥1, we assume that all socioeconomic levels $y_{i}$ are strictly positive. (This excludes the use of data according to which some individuals attain a zero or negative level of socioeconomic achievement. This happens frequently when income data are used for the measurement of socioeconomic status. Instead of income, one might consider using consumption data.) We can likewise define $\mu_{y(\alpha )}=\frac{1}{n}\sum_{i=1}^{n}y_{i}\left(\alpha \right)$. Let us then define the relative position of individual *i* as the ratio of the deviation $d_{i}\left(\alpha \right)=y_{i}\left(\alpha \right)-\mu_{y(\alpha )}$ to the absolute mean deviation $\mu_{\left|d(\alpha )\right|}$, defined as $\mu_{\left|d(\alpha )\right|}=\frac{1}{n}\sum_{i=1}^{n}\left|y_{i}\left(\alpha \right)-\mu_{y(\alpha )}\right|$. The modified weighting function changes the deviation component, but keeps the inequality component unchanged. We therefore arrive at the following definition of the weights:

(20) $$w_{i}^{SL}\left(\alpha \right)=\frac{y_{i}\left(\alpha \right)-\mu_{y(\alpha )}}{\frac{1}{n}\sum_{j=1}^{n}\left|y_{j}\left(\alpha \right)-\mu_{y(\alpha )}\right|}.\frac{\frac{1}{n}\sum_{j=1}^{n}\left|y_{j}-\mu_{y}\right|}{\mu_{y}}$$

with the corresponding bivariate inequality index denoted by SL(α).

It is easy to see that the case α=0 corresponds to our original proposition: no transformation is applied to the socioeconomic levels. As α increases, the weight of the most well-off individual remains positive but decreases in magnitude, whereas the weight of the least well-off individual remains negative but increases in magnitude. What happens to the weights of the other individuals depends on the specific socioeconomic distribution. More and more individuals who initially had a negative weight will get a positive weight, until eventually only the least well-off individual will be the only one with a negative weight. As in the basic case, all the negative weights sum to $-\left(n/2\right)\left(\mu_{\left|d\right|}/\mu_{y}\right)$, and all the positive weights to $\left(n/2\right)\left(\mu_{\left|d\right|}/\mu_{y}\right)$. Since $\mu_{\left|d\right|}$ is at most equal to $2\left(n-1\right)\mu_{y}/n$, it follows that these two amounts are bounded by 1−n and n−1. For high values of α, therefore, the lowest negative weight will tend to $-\left(n/2\right)\left(\mu_{\left|d\right|}/\mu_{y}\right)\geq 1-n$, while all the other weights will be positive and tend to $\left(n/2\right)\left(\mu_{\left|d\right|}/\mu_{y}\right)/\left(n-1\right)\leq 1$.

#### 3.6.4. Assessing Extended Indices

The extended indices proposed above all have the properties of Anonimity, Unit consistency and Neutrality. It is true that the extended rank-dependent indices give greater weight to individuals at the bottom of the distribution, but the effect is a function of the ranks, not the levels, of those individuals. What we have is distributional sensitivity in terms of ranks rather than levels. Strictly speaking, therefore, the extended indices SR(ν) do not have the Sensitivity property.

The extended level-dependent indices SL(α) do have the Sensitivity property, but at the (inevitable) price of losing the Coherence property. Due to the nonlinear character of the SL(α) weighting function, it becomes possible that a transfer $\delta_{y}$ which increases the correlation between the socioeconomic and the health variable does not lead to an increase of the index. The reason is that for α>0, a transfer $\delta_{y}>0$ from individual *i* to individual *j* changes the weights of all individuals, not just those of *i* and *j*. The weight of individual *i* decreases and that of individual *j* increases, while the weights of other individuals may either increase or decrease. In fact, $\mu_{y(\alpha )}$ decreases, which changes all deviations $d_{i}\left(\alpha \right)=y_{i}\left(\alpha \right)-\mu_{y(\alpha )}$. As a result, the absolute mean deviation $\mu_{\left|d(\alpha )\right|}$ also changes. Since it is possible that $\mu_{\left|d\right|}$ also changes, we have three factors which may affect the values of the weights. The weighting function can in fact be written as: $w_{i}^{SL}\left(\alpha \right)=\left(d_{i}\left(\alpha \right)/\mu_{\left|d(\alpha )\right|}\right)\left(\mu_{\left|d\right|}/\mu_{y}\right)$.

Even if we ignore the effects of the changes in the other weights, which will be small, we cannot be sure that the SL(α) index changes in the right direction. In order to have the Coherence property, the value of SL(α) should increase in reaction to a transfer $\delta_{y}>0$ from individual *i* to individual *j*, where *j* is in better health than *i* ($h_{i}<h_{j}$). It could very well be that the decrease of $w_{i}$ is larger than the increase of $w_{j}$, i.e., that $\Delta w_{i}+\Delta w_{j}<0$. In that case, the index will decrease rather than increase if we have $-\Delta w_{j}/\Delta w_{i}<h_{i}/h_{j}$. It seems that only for relatively large values of α, $\Delta w_{j}$ will be substantially smaller than $-\Delta w_{i}$. We can expect, therefore, that for relatively small values of α, the index SL(α) will nearly always change in the right direction in response to a transfer $\delta_{y}$. In any case, the index always reacts appropriately to a transfer $\delta_{h}$ of the health variable. Hence, the price that has to be paid for the introduction of lower-end distributional sensitivity seems relatively modest.

### 3.7. Inverted Indices

#### 3.7.1. Switching Health and Socioeconomic Status

So far, we followed the usual practice of treating socioeconomic status as the weighting variable, and health as the weighted variable (i.e., we have taken z=y and x=h). We have indicated how the weights can be defined in terms of the socioeconomic levels rather than the ranks, and how these weights can be made dependent upon the socioeconomic distribution in order to express concern for the poorer part of the population. However, what if we want to introduce distributional sensitivity to the lower end of the health distribution and express concern for those who are worse-off in terms of health?

In our opinion, the easiest solution consists of turning the approach on its head. We may just as well treat health as the weighting variable, and socioeconomic status as the weighted variable (i.e., z=h and x=y). This can be done both for rank-dependent and for level-dependent indices.

#### 3.7.2. Inverted Rank-Dependent Indices

It is well-known that the Concentration Index has been developed by analogy to the Gini coefficient. The Gini coefficient is a univariate inequality measure which uses both the levels and the ranks of the single variable of interest (e.g., income). The Concentration Index, by contrast, is a bivariate inequality measure which considers the ranks of the socioeconomic variable and the levels of the health variable. However, it is nowhere carved in stone that the socioeconomic variable should enter only through its ranks and the health variable only through its levels, and not the other way around. As a matter of fact, if the health variable is of an ordinal kind, it seems more natural to use the ranks of the health variable and the levels of the socioeconomic variable (see [8]). This leads to a family of indices in which the weighted variable is socioeconomic status and the weights depend on the ranks which individuals occupy in the health distribution. The counterpart of the health Concentration Index would then be the socioeconomic status Concentration Index. It is based on the ‘health rank’ weighting function:

(21) $$w_{i}^{HR}=\frac{2q_{i}-n-1}{n}$$

where $q_{i}$ represents the rank of individual *i* in the health distribution. The associated bivariate inequality index is denoted by HR. Likewise, the extended version of this index, HR(λ), would be based on the following weighting function:

(22) $$w_{i}^{HR}\left(\lambda \right)=\left\{\begin{array}{c} \frac{1+n\left[{\left(\frac{n-q_{i}}{n}\right)}^{\lambda }-{\left(\frac{n-q_{i}+1}{n}\right)}^{\lambda }\right]}{\lambda -1},\;\;\;\;\;\lambda >0,\lambda \neq 1\\ \left(n-q_{i}\right)\ln\left(\frac{n-q_{i}}{n}\right)-\left(n-q_{i}+1\right)\ln\left(\frac{n-q_{i}+1}{n}\right),\;\;\;\;\;\lambda =1 \end{array}\right.$$

with λ as the distributional sensitivity parameter.

#### 3.7.3. Inverted Level-Dependent Indices

As before, we can raise the objection that if we focus on the health ranks only, we throw away a lot of relevant information. Hence, it seems useful to define the weights in terms of the health levels. Following the same reasoning as before, a first possibility is that we use the following level-dependent weighting function:

(23) $$w_{i}^{HL}=\frac{h_{i}-\mu_{h}}{\mu_{h}}$$

which leads to the index HL. If we want to introduce sensitivity to the health distribution, we can do so by first transforming the health levels:

(24) $$h_{i}\left(\beta \right)=\left\{\begin{array}{cc} \frac{h_{i}^{1-\beta }-\beta }{1-\beta } & \left(\beta \neq 1\right)\\ 1+\ln(h_{i}) & \left(\beta =1\right) \end{array}\right.$$

and then using the following weighting function:

(25) $$w_{i}^{HL}\left(\beta \right)=\frac{h_{i}\left(\beta \right)-\mu_{h(\beta )}}{\frac{1}{n}\sum_{j=1}^{n}\left|h_{j}\left(\beta \right)-\mu_{h(\beta )}\right|}.\frac{\frac{1}{n}\sum_{j=1}^{n}\left|h_{j}-\mu_{h}\right|}{\mu_{h}}$$

This yields the index HL(β). Higher values of β make the index more sensitive to the lower end of the health distribution.

## 4. Materials and Methods III: Rank-Dependent vs. Level-Dependent Indices

### 4.1. A Comparison of the Indices

It may be useful to sum up how we suggest to move from rank-dependent indices (SR, SR(ν), HR, HR(λ)) to level-dependent indices (SL, SL(α), HL, HL(β)). For each rank-dependent index we have constructed a level-dependent alternative, as indicated in Table 1:

If the levels of both the socioeconomic status and the health variable are known, there is in general no reason why one should stick to rank-dependent indices. Only when some of the levels of the weighting variable are equal to zero, the extended versions of the level-dependent indices cannot be computed for α,β≥1.

The basic versions SR, SL, HR and HL are all special cases of the extended versions, taking ν,λ=2 and α,β=0. Since rank-dependent weights differ from level-dependent weights (except by fluke), the values of the associated indices are also different. Put differently, in general SR is unrelated to SL, and HR to HL. Likewise, there is no reason why the two rank-dependent indices SR and HR should be related to one another. However, the same does not hold with respect to the two level-dependent indices. This can be illustrated by expressing SL and HL in terms of covariances. In fact, we have:

(26) $$SL=\frac{1}{n}\sum_{i=1}^{n}\frac{y_{i}-\mu_{y}}{\mu_{y}}h_{i}=\frac{Cov(y,h)}{\mu_{y}}$$

(27) $$HL=\frac{1}{n}\sum_{i=1}^{n}\frac{h_{i}-\mu_{h}}{\mu_{h}}y_{i}=\frac{Cov(y,h)}{\mu_{h}}$$

From these expressions we can derive that the relative versions of the two indices are equal to one another:

(28) $$\frac{SL}{\mu_{h}}=\frac{HL}{\mu_{y}}=\frac{Cov(y,h)}{\mu_{y}\mu_{h}}$$

Expression (28) reveals that the two basic level-dependent indices are closely related to the class of covariance-based indices derived axiomatically by Bidard [33], and to the Atkinson-type index derived by Erreygers [34]. There is also a link with the κ index proposed by Abul Naga and Geoffard [18]: 365, for a specific choice of their parameters α and β; in fact, when α=β=1, it turns out that we have $\kappa -1=SL/\mu_{h}=HL/\mu_{y}$. (This follows from the definition $\kappa =\frac{n\sum_{i=1}^{n}y_{i}^{\alpha }h_{i}^{\beta }}{\sum_{i=1}^{n}y_{i}^{\alpha }\sum_{i=1}^{n}h_{i}^{\beta }}$ and from the equality $Cov\left(y,h\right)=\frac{1}{n}\sum_{i=1}^{n}y_{i}h_{i}-\mu_{y}\mu_{h}$.) Zhong [19] has applied κ, or more precisely ln(κ), to study income-related health inequality in China, but it should be noted that his values of α and β differ from ours.

### 4.2. A Comparison of the Weighting Functions

We can shed additional light on the difference between rank-dependent and level-dependent indices by comparing the weighting functions which characterise the two types of indices. We focus here on the case where the weighting variable is socioeconomic status.

The two weighting functions, $w_{i}^{SR}\left(\nu \right)$ for the rank-dependent indices and $w_{i}^{SL}\left(\alpha \right)$ for the level-dependent indices, can be visualised in two ways. The first consists of mapping them in function of the ranks $r_{i}$, or more precisely, in function of the fractional ranks $\left(2r_{i}-1\right)/\left(2n\right)$, for successive values of *i* (i.e., 1/(2n),3/(2n),…,(2n−1)/(2n)). In this way, we can examine how the weights change as one moves up or down the rank order. The second way consists of mapping them in function of the levels $y_{i}$, again for successive values of *i* (i.e., $y_{1},y_{2},\ldots ,y_{n}$). This allows us to track the evolution of the weights for changing values of the socioeconomic variable.

**Figure 1:**
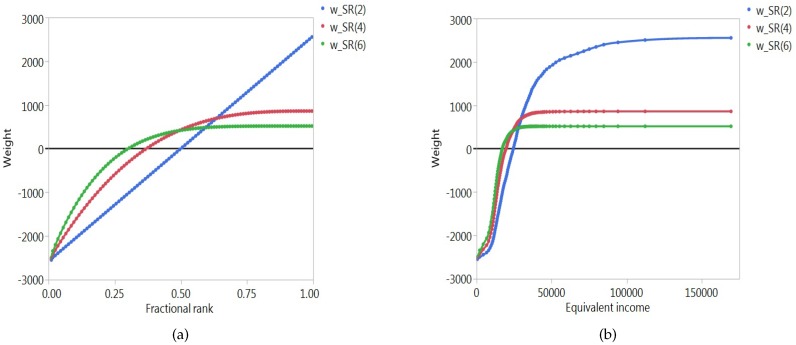
Rank-dependent weighting functions for the SR(2), SR(4) and SR(6) indices in terms of the fractional ranks (**a**) and the equivalent incomes (**b**) for The Netherlands.

**Figure 2:**
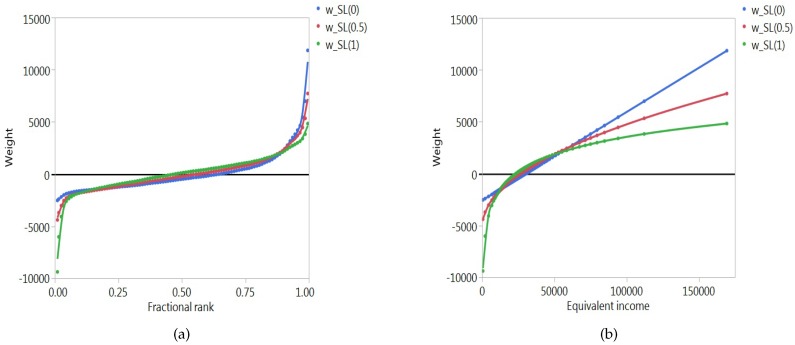
Level-dependent weighting functions for the SL(0), SL(0.5) and SL(1) indices in terms of the fractional ranks (**a**) and the equivalent incomes (**b**) for The Netherlands.

Figure 1 and Figure 2 illustrate the situation for a typical country. The country we have chosen is The Netherlands. In the following section we provide more details on the socioeconomic variable (equivalent individual income) and the health variable (well-being). Instead of representing each individual separately, we have grouped individuals into percentiles. Each dot represents approximately 1% of the population. Panel a of Figure 1 represents the rank-dependent weighting function in terms of the fractional ranks, for three different values of the parameter ν (ν=2,4,6). For ν=2, the weighting function is linear, as can be seen from expression (13). For higher values of ν, the curve becomes steeper at the left and flatter at the right, and the point of intersection with the horizontal axis shifts to the left. A rather different picture emerges if one represents the level-dependent weighting function in the same way, again for three different values of the parameter α ($\alpha =0,\frac{1}{2},1$), as can be seen from Panel a of Figure 2. It is striking, for instance, that for relatively low values of α the weighting function steeply rises on the right. This occurs when those who are well-off in socioeconomic terms have high levels of the socioeconomic variable. Panel b of Figure 1 represents the rank-dependent weighting function in terms of the levels of the socioeconomic variable. For every value of the parameter ν the weighting function has an elongated *S*-shape. The corresponding representation of the level-dependent weighting function can be found in Panel b of Figure 2. For α=0, the weighting function is linear (cf. expression (16)), and for higher values of α, the function becomes steeper at the left and flatter at the right, with the point of intersection with the horizontal axis moving to the left.

**Figure 3:**
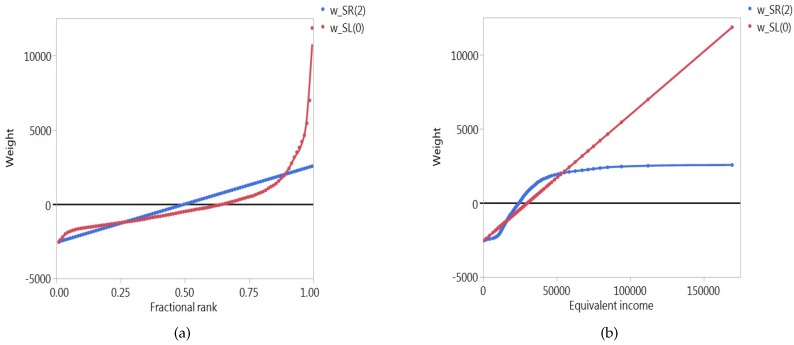
Overlay rank- and level-dependent weighting functions for the basic SR and SL indices in terms of the fractional ranks (**a**) and the equivalent incomes (**b**) for The Netherlands.

Figure 3 highlights the differences between the basic versions of the two types of indices. In this specific example, the level-dependent index gives relatively more weight to the highest ranked individuals, as can be seen from Panel a. Or, to put it differently, the rank-dependent index gives relatively less weight to individuals who attain a high socioeconomic level, as shown by Panel b.

## 5. Results

In order to explore whether it makes a difference to use level-dependent rather than rank-dependent indices, we conduct a comparative study on the relation between income and well-being. This study allows us also to find out whether a change in the sensitivity to inequality, either with regard to the socioeconomic distribution or with regard to the health distribution, affects the results. We look at the ranking of a given set of countries according to the different rank-dependent and level-dependent indices we have encountered above. For each of the parameters we take three different values: ν,λ=2,4,6, and $\alpha ,\beta =0,\frac{1}{2},1$. All in all, we therefore have 12 indices we can use to rank countries.

### 5.1. Data

Our data come from SHARE. We use data from Wave 4, which are derived from surveys taken in 2010–2011 (more details can be found in [35]). Our socioeconomic variable is based on “total household income” (thinc). The health variable we have selected is the “quality of life and well-being index” (casp), known as CASP-12. This is a variable derived from scores on 12 dimensions of well-being [36]. Data are available for 16 countries: Austria, Belgium, Czechia, Denmark, Estonia, France, Germany, Hungary, Italy, The Netherlands, Poland, Portugal, Slovenia, Spain, Sweden and Switzerland.

The SHARE data are available at the level of the household (income) and of the individual (well-being). Since income data are often approximate, we have decided to adopt a procedure to smoothen the data. This also helps to avoid that a few observations at the extremes, i.e., very low and very high incomes, distort the results. First, we translated household incomes into equivalent individual incomes using the Organisation for Economic Co-operation and Development (OECD) equivalence scale, which consists of dividing the household income by the square root of the number of household members. Next, we ranked individuals according to their equivalent income and grouped them into percentiles taking into account their sample weights, i.e., we partitioned the population into 100 groups of equal weight. As sample weights, we used the “calibrated cross-sectional weight wave 4” (ciw_w4). Because of the irregular nature of the sample weights, it occurred that some groups are slightly larger or slightly smaller than 1%. For each percentile we then calculated the mid-interval value of the equivalent income. This means that if in a given percentile $y_{\min}$ is the lowest and $y_{\max}$ the highest equivalent individual income, then the mid-interval value is equal to $y_{\min}+(y_{\max}-y_{\min)}/2$. Finally, this mid-interval income was assigned to every individual of that percentile. In a few countries of our database, a relatively large fraction of the population reported a zero income. For these countries, our procedure would lead to a zero mid-interval income for the first percentile, and maybe also for the second, the third and so on. Suppose that the *k*-th percentile is the first percentile with a non-zero mid-interval income. If k>1, we decided to assign to all individuals of percentiles 1 to *k* the average income of the percentiles 1 to *k* taken together. We applied this procedure to Germany, Hungary, Slovenia and Spain (k=2), and to Italy and Portugal (k=4). In this way, we always assign a positive income to all individuals. It must be noted, however, that when the fraction of the population with a very low assigned income is considerable, our results may be biased, especially for values of α greater than or equal to 1. A thorough discussion on the sensitivity of inequality measures to very high and very low incomes can be found in [37].

The well-being variable CASP-12 is a bounded variable, which varies between a lower bound of 12 and an upper bound of 48. Its value is calculated as the total score on 12 questions covering different dimensions of well-being: “Respondents were asked, how often they experience certain feelings and situations on a 4-point scale ranging from ‘never’ to ‘often’.” ([36]: 200) The higher the CASP-12 score, the better the quality of life of a person is supposed to be. A value of 39 or higher is considered to indicate a very high quality of life, a value of 37 or 38 a high quality of life, a value of 35 or 36 a moderate quality of life, and a value below 35 a low quality of life. A few key descriptive statistics of our database can be found in Table 2.

### 5.2. Findings

Table 3 reports the results of the 12 indices. Given that the individuals in our database do not all have the same sample weight, and that some individuals are tied (i.e., attain the same level of the weighting variable), the formulas of the text need to be modified. The Appendix provides more details on the adjusted formulas. Since our health variable is bounded, we use the absolute version of the SR and SL indices. By contrast, given that our socioeconomic variable is unbounded, we use the relative version of the HR and HL indices. As far as the rank-dependent indices are concerned, these choices are motivated by the arguments advanced by Erreygers and Van Ourti [27]. It seems reasonable to apply the same choices to the level-dependent indices.

Table 4 shows the rankings of countries according to all 12 indices. The first observation is that the choice of the index matters. The ranking of countries according to the SR indices is different from that according to the SL indices, and these are in turn different from the rankings according to the HR and the HL indices. While the SR indices identify Portugal, Denmark and Czechia as the countries with the lowest degree of pro-rich inequality, and Poland and Germany as the countries with the highest, the SL indices reveal a different pattern: at the lower end we find Denmark, Sweden and The Netherlands, and at the higher end Italy, Estonia and Slovenia. As pointed out above, for some countries the results may be biased for values of α≥1. The results for α=1 must therefore be treated with caution. Secondly, the rankings according to the HR and HL indices are more stable than these according to the SR and SL indices. This is probably due to the fact that in general, the health variable is less unequally distributed than the socioeconomic variable. As a result, a change in the distributional parameter λ or β when well-being is the weighting variable, tends to have less effect than a change in the parameter ν or α when income is the weighting variable. In what follows, we concentrate on the two types of indices for which income serves as the weighting variable.

**Figure 4:**
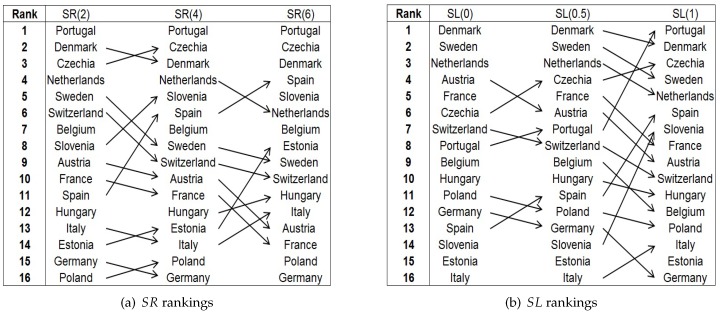
Visualization of the effect of changes of ν(ν=2,4,6) on the SR ranking of countries (**a**) and of α(α=0,0.5,1) on the SL ranking of countries (**b**).

Figure 4 illustrates the effect of changes in the distributional parameter on the ranking of countries. In every column the country with the lowest degree of pro-rich inequality is on top, and the country with the highest at the bottom. Panel a shows the effect of a change in ν on the ranking of countries according to the rank-dependent indices. As ν increases, countries like Austria, France, Sweden and Switzerland drop down, while Estonia and Spain make gains. Panel b shows the effect of a change in α. Countries like Austria, Germany, Poland and Switzerland see their position in the ranking according to the level-dependent indices worsen, while Czechia and Portugal move up. On the whole, one might say that the rankings are different, that they are influenced by changes in the distributional parameter, but that the effects are limited. A look at the correlation coefficients (Table 5, Pearson’s or Spearman’s rank correlations) reveals that the three SR indices are strongly positively correlated among themselves, especially SR(2) and SR(4), and SR(4) and SR(6). The same seems to hold for the three SL indices, although to a lesser extent for SL(1). This may be due to the presence of a significant amount of individuals with very low incomes. The correlations between the SR and the SL indices are all positive, but some of them are very low and even insignificant at the 5% level, namely those between SR(6) and SL(0), between SR(6) and SL(0.5), and between SR(4) and SL(0).

**Figure 5:**
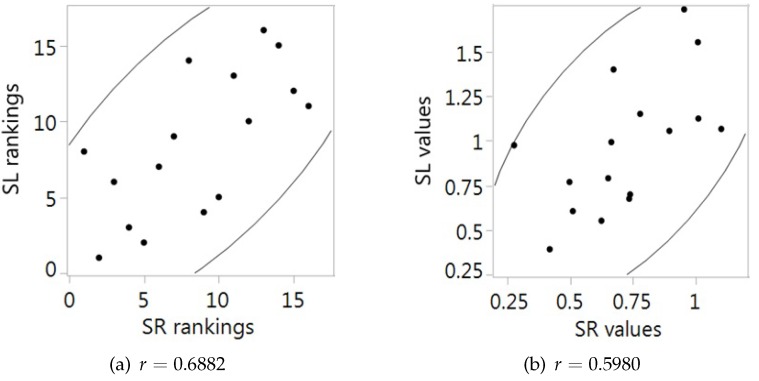
Scatter plots showing the correlation between the basic SR and SL rankings (**a**) and values (**b**) for the 16 countries.

Perhaps the best way of comparing the rank-dependent and the level-dependent indices is to focus on the two basic versions of the indices. The two scatter diagrams of Figure 5 visualise the difference between SR and SL, both in terms of the rankings of countries (Panel a) and in terms of the values of the indices (Panel b). Since the two indices use the same information but process it differently, the results are similar but by no means identical. The two rankings are positively correlated, and so are the two values, but in both cases the correlation is far from perfect. For the rankings, the coefficient of correlation *r* is equal to 0.6882, and for the values, it is equal to 0.5980. Therefore, it does make a difference which index you use. It may very well happen that if one compares the situation in a given country at two different moments of time, one of the indices measures an increase and the other a decrease. Since our empirical application is limited to one year only, it cannot be used to illustrate this point.

## 6. Discussion

### 6.1. Theoretical Issues

Above we have argued that, in our view, there are good reasons to move away from the rank-dependent indices which dominate the literature on the measurement of socioeconomic inequality of health. Moreover, it has been shown recently that the application of rank-dependent indices to population-weighted samples may produce severely biased results; for more details, see [38]. The level-dependent indices we propose as alternatives are, however, not the only ones which can be thought of. To begin with, surely there exist other ways of defining level-dependent weights than the two-part procedure advocated here. Our level-dependent weights consist of a ‘positional’ part which determines the position of an individual relative to others, and an ‘inequality’ part which determines the sum of the absolute values of all the weights. Even if one agrees with that procedure, alternative roads can be followed. In our construction, the positional part is either directly proportional to the level of the weighting variable, or proportional to a transformation of this level. One may think of other forms of transformation than the one we have introduced above, i.e., based upon the isoelastic function. Likewise, the specific inequality measure we have used in our formulas, i.e., the relative mean deviation, is by no means the only one available. Alternative measures such as the Gini coefficient or the Theil index could be used just as well. The advantage of the relative mean deviation, however, is that it allows us to establish a direct connection between the level-dependent bivariate inequality measure and the standard statistical concept of covariance.

To sum up, what we propose here is *one* way of defining a family of level-dependent bivariate inequality measures. The specific form we have chosen gives rise to a very simple weighting function in the basic case, while allowing the expression of distributional sensitivity in the extended case. The specification of additional desired properties might further refine the set of acceptable indices. A systematic comparison of the indices introduced here and the indices of association proposed by Abul Naga and Geoffard [18] and used by Zhong [19] seems called for. Such a comparison will yield additional insights into the properties of the indices; obviously, that kind of information will be of great use for practitioners.

### 6.2. Empirical Issues

As far as our empirical application is concerned, the importance of it should not be exaggerated. We have conceived it as an illustration of the potential differences between rank-dependent and level-dependent bivariate inequality measures, not as an in-depth study of the relationship between income and well-being.

The data we have used are far from perfect. Perhaps the most important criticism which can be made of our study is that we treat the CASP-12 variable as a ratio-scale measure of well-being. It would be entirely legitimate to challenge this assumption. If it were a cardinal instead of a ratio-scale variable, it would be inappropriate to use the relative mean deviation as a measure of the inequality in well-being, and therefore to define weights in terms of this variable. This implies that the HL indices, which are based on weights dependent on the levels of the well-being variable, would be unreliable. For this reason, we have chosen to focus on the SL indices, with weights dependent on the levels of income, in our presentation of the results. With regard to the SL indices, we have already pointed out that the occurrence of zero or very low incomes poses a problem for values of the distributional sensitivity parameter greater than or equal to 1.

## 7. Conclusions

When it comes to the measurement of bivariate inequality, and in particular of the socioeconomic inequality of health, we believe it is time to move from rank-dependent to level-dependent indicators. Our choice is based upon the value judgment that it is preferable to use more rather than less information, unless there are solid grounds to argue that the additional information is obsolete or irrelevant. Rank-dependent indicators ignore quite a lot of valuable information on one of the two dimensions which are being considered. Level-dependent indicators, by contrast, do take that information into account. Moreover, just as rank-dependent indicators, level-dependent indicators can incorporate distributional sensitivity.

But does this matter? In this paper we have shown that it does: our empirical study yielded different results for different indices. When comparisons are made between countries or over time, rank-dependent and level-dependent indicators do not necessarily produce the same outcome. Although rank-dependent indicators have been used for a long time now and remain popular in empirical research on the socioeconomic inequality of health, we are convinced that level-dependent indicators are to be preferred.

## Figures and Tables

**Table 1:** Indices of socioeconomic inequality of health.

Weighting Variable	Version	Rank-Dependent	Level-Dependent
Socioeconomic status	$\begin{array}{c} \mathrm{Basic}\\ \mathrm{Extended} \end{array}$	$\begin{array}{c} SR\\ SR(\nu ) \end{array}$	$\begin{array}{c} SL\\ SL(\alpha ) \end{array}$
Health	$\begin{array}{c} \mathrm{Basic}\\ \mathrm{Extended} \end{array}$	$\begin{array}{c} HR\\ HR(\lambda ) \end{array}$	$\begin{array}{c} HL\\ HL(\beta ) \end{array}$

**Table 2:** Descriptive statistics of the variables under study for the 16 countries.

	*n*	Equivalent Income			Health		
		Mean	Median	RMD	Mean	Median	RMD
Austria	4,807	24,054	18,809	0.5488	39.60	41	0.1206
Belgium	4,787	49,319	24,903	0.8441	37.22	38	0.1343
Czechia	5,497	7,641	6,355	0.5439	34.79	35	0.1350
Denmark	2,110	32,997	29,258	0.4172	40.72	41	0.0902
Estonia	5,997	5,919	4,384	0.5978	35.12	36	0.1579
France	5,011	26,258	21,240	0.5165	37.96	39	0.1254
Germany	1,469	22,489	17,647	0.5564	38.22	39	0.1232
Hungary	2,901	5,261	4,307	0.5308	34.00	34	0.1650
Italy	3,365	17,455	12,407	0.6451	33.70	34	0.1564
Netherlands	2,575	30,248	24,272	0.5224	40.67	41	0.0944
Poland	1,629	4,279	3,646	0.5031	35.56	36	0.1548
Portugal	1,887	11,163	5,409	0.9698	31.87	32	0.1171
Slovenia	2,574	18,890	9,890	0.8596	39.20	40	0.1217
Spain	3,252	13,417	10,104	0.6152	35.81	36	0.1481
Sweden	1,860	31,016	26,497	0.4769	38.79	39	0.1054
Switzerland	3,536	70,520	51,656	0.6191	40.62	42	0.0967

RMD: Relative Mean Deviation.

**Table 3:** Absolute SR and SL indices and relative HR and HL indices for the 16 countries.

	SR(ν)			SL(α)			HR(λ)			HL(β)		
	ν=2	ν=4	−ν=6	α=0	α=0.5	α=1	λ=2	λ=4	λ=6	β=0	β=0.5	β=1
Austria	0.7365	0.4685	−0.3369	0.6752	0.7829	0.8492	0.0639	0.0422	0.0319	0.0171	0.0173	0.0176
Belgium	0.6651	0.4125	−0.2808	0.9930	1.0287	1.0816	0.0984	0.0575	0.0406	0.0267	0.0266	0.0263
Czechia	0.4981	0.2357	−0.1469	0.7689	0.6566	0.4165	0.0769	0.0479	0.0350	0.0221	0.0223	0.0226
Denmark	0.4191	0.2580	−0.1909	0.3913	0.3876	0.3830	0.0430	0.0341	0.0275	0.0096	0.0099	0.0102
Estonia	1.0106	0.4995	−0.3076	1.5543	1.4020	1.1870	0.1340	0.0776	0.0548	0.0443	0.0441	0.0439
France	0.7402	0.4798	−0.3512	0.6993	0.7717	0.8358	0.0683	0.0447	0.0325	0.0184	0.0187	0.0190
Germany	1.0125	0.6871	−0.5309	1.1255	1.2585	1.5130	0.1103	0.0720	0.0538	0.0295	0.0299	0.0303
Hungary	0.8968	0.4949	−0.3291	1.0562	1.0384	0.9885	0.0875	0.0530	0.0401	0.0311	0.0313	0.0317
Italy	0.9558	0.5014	−0.3297	1.7387	1.5127	1.1600	0.1585	0.0854	0.0578	0.0516	0.0508	0.0500
Netherlands	0.5113	0.3189	−0.2374	0.6054	0.6028	0.5882	0.0701	0.0480	0.0361	0.0149	0.0151	0.0154
Poland	1.1040	0.6108	−0.4024	1.0670	1.1070	1.1317	0.0919	0.0596	0.0448	0.0300	0.0304	0.0307
Portugal	0.2770	0.0316	−0.0291	0.9755	0.8013	0.1443	0.1300	0.0652	0.0387	0.0306	0.0298	0.0289
Slovenia	0.6740	0.3700	−0.2367	1.4010	1.3195	0.7843	0.1460	0.0783	0.0542	0.0357	0.0356	0.0353
Spain	0.7801	0.3826	−0.2313	1.1517	1.0469	0.7481	0.1027	0.0666	0.0486	0.0322	0.0324	0.0327
Sweden	0.6256	0.4276	−0.3156	0.5513	0.5824	0.5869	0.0606	0.0408	0.0314	0.0142	0.0143	0.0144
Switzerland	0.6527	0.4366	−0.3258	0.7903	0.8608	0.9780	0.0838	0.0568	0.0446	0.0195	0.0199	0.0204

**Table 4:** Rankings based on the absolute SR and SL indices and relative HR and HL indices for the 16 countries.

	SR(ν)			SL(α)			HR(λ)			HL(β)		
	ν=2	ν=4	ν=6	α=0	α=0.5	α=1	λ=2	λ=4	λ=6	β=0	β=0.5	β=1
Austria	19	10	13	14	16	19	13	13	13	14	14	14
Belgium	17	17	17	19	19	12	10	19	19	18	18	18
Czechia	13	12	12	16	14	13	16	15	15	17	17	17
Denmark	12	13	13	11	11	12	11	11	11	11	11	11
Estonia	14	13	18	15	15	15	14	14	15	15	15	15
France	10	11	14	15	15	18	14	14	14	15	15	15
Germany	15	16	16	12	13	16	12	13	13	19	10	10
Hungary	12	12	11	10	10	11	18	17	18	12	12	12
Italy	13	14	12	16	16	14	16	16	16	16	16	16
Netherlands	14	14	16	13	13	15	15	16	16	13	13	13
Poland	16	15	15	11	12	13	19	10	11	10	11	11
Portugal	11	11	11	18	17	11	13	11	17	11	19	19
Slovenia	18	15	15	14	14	17	15	15	14	14	14	14
Spain	11	16	14	13	11	16	11	12	12	13	13	13
Sweden	15	18	19	12	12	14	12	12	12	12	12	12
Switzerland	16	19	10	17	18	10	17	18	10	16	16	16

**Table 5:** Correlation matrix of country rankings based on the absolute SR and SL indices.

	SR(2)	SR(4)	SR(6)	SL(0)	SL(0.5)	SL(1)
SR(2)	1.0000					
SR(4)	0.9118	1.0000				
SR(6)	0.7529	0.9147	1.0000			
SL(0)	0.6882	${0.4706\;}^{§}$	$0.1735^{§}$	1.0000		
SL(0.5)	0.7647	0.5971	${0.3324\;}^{§}$	0.9765	1.0000	
SL(1)	0.8735	0.9000	0.7500	0.6441	0.7559	1.0000

All correlations are significant at the 1% level, except for the ones indicated by a ‘§’ sign which are insignificant at the 5% level.

## Appendix. Sample Weights and Ties

This appendix describes how the weights $w_{i}$ must be defined when working with datasets in which not all individuals have the same sample weight, and when there are ties between individuals with respect to the weighting variable. We focus here on the case in which the socioeconomic variable (income, for simplicity) serves as the weighting variable. It is straightforward to extend the formulas to the case in which the health variable serves as the weighting variable.

We assume that our dataset consists of individuals 1,2,…,n, ranked according to their individual income, i.e., $y_{1}\leq y_{2}\leq \ldots \leq y_{n}$. Let $s_{i}$ be the sample weight of individual *i*. We assume that $\sum_{i=1}^{n}s_{i}=1$.

If there are ties, we define *K* groups of individuals $G_{1},G_{2},\ldots ,G_{K}$ such that $y_{G_{1}}<y_{G_{2}}<\ldots <y_{G_{K}}$. The sample weight of group $G_{J}$ is $s_{G_{J}}=\sum_{i\in G_{J}}s_{i}$. The cumulative sample weight of group $G_{J}$ is defined by the recursive formula $c_{G_{J}}=c_{G_{J-1}}+s_{G_{J}}$, where we take $c_{G_{0}}=0$.

### Rank-Dependent Weights

Let individual *i* belong to group $G_{J}$. Then for ν>0,ν≠1 the weight of this individual is equal to:

(A1) $$w_{i}^{SR}\left(\nu \right)=\left(\frac{1}{\nu -1}\right)n\left(s_{i}+\left(\frac{s_{i}}{s_{G_{J}}}\right)\left[{\left(1-c_{G_{J}}\right)}^{\nu }-{\left(1-c_{G_{J-1}}\right)}^{\nu }\right]\right)$$

and for ν=1:

(A2) $$w_{i}^{SR}\left(\nu \right)=\left(\frac{s_{i}}{s_{G_{J}}}\right)n\left[\left(1-c_{G_{J}}\right)\ln\left(1-c_{G_{J}}\right)-\left(1-c_{G_{J-1}}\right)\ln\left(1-c_{G_{J-1}}\right)\right]$$

### Level-Dependent Weights

When working with level-dependent weights, there is no need to consider group weights. We do have to modify the definitions of the mean income $\mu_{y(\alpha )}$ and of the absolute mean deviation $\mu_{\left|d(\alpha )\right|}$. The mean weighted income is now $\mu_{y(\alpha )}=\sum_{i=1}^{n}s_{i}y_{i}\left(\alpha \right)$, and the absolute mean weighted deviation $\mu_{\left|d(\alpha )\right|}=\sum_{i=1}^{n}s_{i}\left|y_{i}\left(\alpha \right)-\mu_{y(\alpha )}\right|$. Taking α=0, we obtain $\mu_{y}=\sum_{i=1}^{n}s_{i}y_{i}$ and $\mu_{\left|d\right|}=\sum_{i=1}^{n}s_{i}\left|y_{i}-\mu_{y}\right|$. Using these definitions, the weight of individual *i* is equal to:

(A3) $$w_{i}^{SL}\left(\alpha \right)=\frac{ns_{i}\left[y_{i}\left(\alpha \right)-\mu_{y(\alpha )}\right]}{\sum_{j=1}^{n}s_{j}\left|y_{j}\left(\alpha \right)-\mu_{y(\alpha )}\right|}.\frac{\sum_{j=1}^{n}s_{j}\left|y_{j}-\mu_{y}\right|}{\mu_{y}}$$
